# Molecular basis for human aquaporin inhibition

**DOI:** 10.1073/pnas.2319682121

**Published:** 2024-02-06

**Authors:** Peng Huang, Hannah Åbacka, Carter J. Wilson, Malene Lykke Wind, Michael Rűtzler, Anna Hagström-Andersson, Pontus Gourdon, Bert L. de Groot, Raminta Venskutonytė, Karin Lindkvist-Petersson

**Affiliations:** ^a^Department of Experimental Medical Science, Lund University, Lund 22184, Sweden; ^b^Computational Biomolecular Dynamics Group, Department of Theoretical and Computational Biophysics, Max Planck Institute for Multidisciplinary Sciences, 37077 Gottingen, Germany; ^c^Department of Biomedical Sciences, Copenhagen University, DK-2200 Copenhagen N, Denmark; ^d^ApoGlyx, Lund 22381, Sweden; ^e^Division of Biochemistry and Structural Biology, Department of Chemistry, Lund University, Lund 22100, Sweden; ^f^Department of Laboratory Medicine, Division of Clinical Genetics, Lund University, Lund 22184, Sweden; ^g^Lund Institute of Advanced Neutron and X-Ray Science, Lund 22370, Sweden

**Keywords:** aquaporin, AQP7, inhibitor, cryo-EM structure, leukemia

## Abstract

Cancer invasion and metastasis are known to be potentiated by the expression of aquaporins (AQPs). Likewise, the expression levels of AQPs have been shown to be prognostic for survival in patients and have a role in tumor growth, edema, angiogenesis, and tumor cell migration. Thus, AQPs are key players in cancer biology and potential targets for drug development. Here, we present the single-particle cryo-EM structure of human AQP7 at 3.2-Å resolution in complex with the specific inhibitor compound Z433927330. The structure in combination with MD simulations shows that the inhibitor binds to the endofacial side of AQP7. In addition, cancer cells treated with Z433927330 show reduced proliferation. The data presented here serve as a framework for the development of AQP inhibitors.

## Significance

Aquaporins (AQPs) are membrane proteins facilitating flux of water and/or small neutral solutes like glycerol across membranes. Drugs targeting AQPs have broad potential clinical applications, including cancer, obesity, and skin diseases among others. Here, we report the structure of an AQP bound to a drug-like small-molecule compound and show inhibition of leukemia cell proliferation in the presence of the studied inhibitor. The structural analysis in combination with functional data presented here serves as a framework for the development of AQP inhibitors. As AQP deletion has been shown to be beneficial in different types of cancers as well as other diseases, the inhibition mechanism proposed provides a molecular basis for therapeutic strategies in cancer.

Aquaporins (AQPs) are integral membrane proteins facilitating the flux of water (orthodox AQPs) and/or small neutral solutes such as glycerol, arsenite, and urea (aquaglyceroporins) across the lipid bilayer of cells. Thirteen types of human AQPs have been identified (abbreviated AQP0-12) and are distributed in specific cell types across a range of different organs and tissues. AQP expression has been linked to multiple pathologies including cancer, fluid dysregulation, edema, obesity, glaucoma, skin diseases, and other pathologies (1), thus therapeutic targeting of AQPs has broad potential for clinical applications. Several compounds have been developed for AQP inhibition targeting both orthodox AQPs and aquaglyceroporins (2). Nevertheless, the molecular basis for inhibition is largely unknown as no three-dimensional structures of AQPs in complex with inhibitors are available. This also hampers the possibility to execute structure-based drug design for the AQP family.

The human genome encodes four aquaglyceroporins AQP3, AQP7, AQP9, and AQP10 and recently AQP7 was identified as a novel regulator of breast cancer (3). Commending this AQP as a cancer-specific therapeutic target, AQP7 was prognostic for overall survival in patients and decreased expression caused a reduction of primary tumors and metastasis in mouse breast cancer models (3). Here, we report the single-particle cryo-EM structure of human AQP7 at 3.2 Å overall resolution in complex with the specific AQP7 inhibitor Z433927330 (4). The structure in combination with molecular dynamics (MD) simulations show that the inhibitor binds to the endofacial side of AQP7 and hydrogen bonds to the backbone of loop B as well as the Gln183 side chain of transmembrane segment 4. Moreover, a cancer cell line expressing AQP7 treated with Z433927330 has decreased proliferation compared to control, suggesting that AQP7 is a cancer susceptibility factor that could be further explored as a therapeutic target.

## Results

### Overall Structure of Human AQP7 in Complex with Inhibitor Z433927330.

**Fig. 1.**
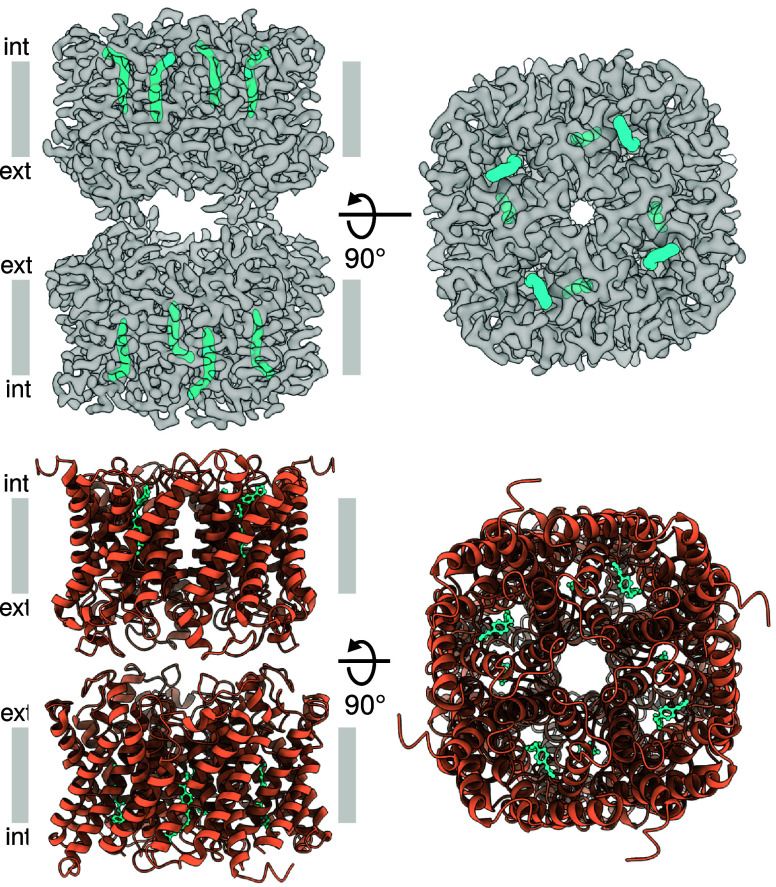
Structural overview of the inhibitor in complex with AQP7 adopting two adhering tetramers. *Upper*: Cryo-EM map of the complex with D4 symmetry applied with AQP7 in gray and the inhibitor in cyan. *Lower*: Cryo-EM model of the complex with AQP7 in deep orange and the inhibitor in cyan.

To facilitate structure-based drug design, the three-dimensional structure of the target protein in complex with a potential drug candidate is required. To that end, human AQP7 was expressed, purified, and subjected to single-particle cryo-EM analyses for structure determination. Protein purification was performed as previously described (5) and two peaks were detected in size-exclusion chromatography, suggesting that different oligomeric species are present in solution (*SI Appendix*, Fig. S1*A*). Prior to vitrification, the previously reported AQP7-specific inhibitor, Z433927330 (4), was added to the protein sample. Single-particle cryo-EM micrographs and related two-dimensional (2D) class averages indicated that AQP7 protein particles can form dimers of tetramers, similar to what was previously observed for the glycerol-bound AQP7 cryo-EM structure (hereafter referred to as AQP7_g_) (5) (*SI Appendix*, Figs. S1*B* and S2). The collected data were processed for model building to a final map at 3.2-Å overall resolution as achieved using D4 symmetry (*SI Appendix*, Table S1 and Fig. S2). As seen for AQP7_g_, the AQP7 with inhibitor (hereafter called AQP7_i_) adopts an octameric configuration with individual glycerol channels forming two adhering tetramers, which interact via their extracellular C loops (Fig. 1). The model shows the typical AQP fold with six transmembrane segments and two half helices located on loop B and loop E (Fig. 1 and *SI Appendix*, Fig. S3). Furthermore, the conserved aromatic/arginine selectivity filter (ar/R) and NPA-motifs (NAA and NPS in AQP7), characteristics of the AQP family, are clear in the model. An unambiguous inhibitor density is observed in the glycerol channel (Fig. 1), showing an extended shape with the kinked end exposed to the intracellular side (Fig. 1). The density is reaching two-thirds into the pores, which is significantly different from the previously reported glycerol densities for AQP7_g_ (*SI Appendix*, Fig. S4). Such binding does not leave any space for glycerol molecules to be present, and thus, we only observe the density corresponding to the inhibitor in the channel (*SI Appendix*, Fig. S4).

### The Inhibition Mechanism of the Substrate Permeability.

**Fig. 2.**
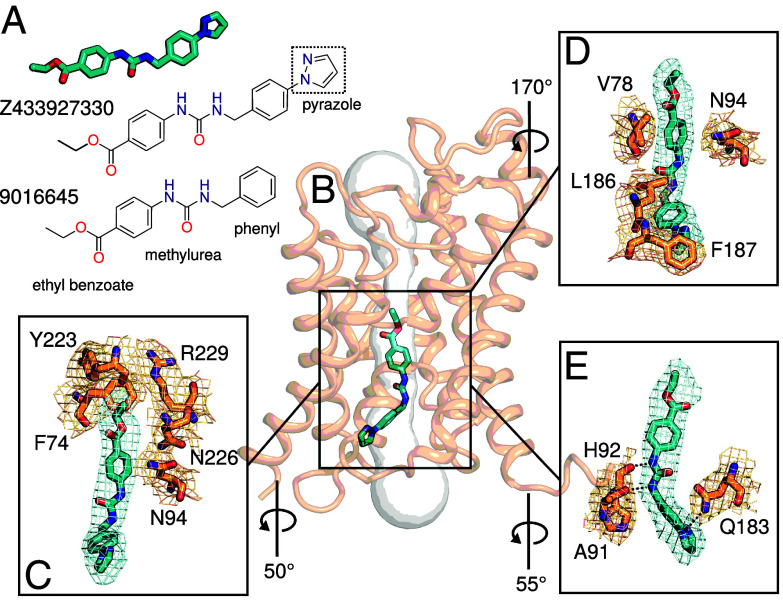
Inhibitor Z433927330 binds in the channel of AQP7. (*A*) Chemical structures of compounds Z433927330 and 9016645. The pyrazole group is indicated. (*B*) AQP7 cryo-EM model shown as cartoon (brown) in complex with the inhibitor in sticks, colored cyan. Software HOLE calculation of the pore in the absence of the inhibitor represented as gray surface. (*C*) Cryo-EM density for the inhibitor and residues in the ar/R selective filter (Phe74, Tyr223 and Arg229) and the NPA motif (Asn94 and Asn226) shown in sticks. (*D*) CH/NH–π interaction between inhibitor and Val78/Asn94. (*E*) H-bonds between the inhibitor and Ala91, His92 and Gln183 are shown as black dashed lines. Cryo-EM densities for inhibitor and residues in all panels are shown as surfaces at the same contour level.

The compound, Z433927330 (Fig. 2*A*), was previously reported to be an inhibitor of AQP7 activity with an IC_50_ ~ 0.2 μM (4). The inhibitor fits into the glycerol channel of AQP7 by binding near the ar/R filter (composed of Phe74, Tyr223, and Arg229) and extends to the endofacial side of the pore (Fig. 2 *B* and *C*). The ethyl-benzoate group is accommodated in the wider pore area in the vicinity of the NPA motifs (Asn94 and Asn226) (Fig. 2 *B* and *C*). There is a possibility for the aromatic ring of the benzoate moiety to participate in NH–π and CH–π interactions with Asn94 and Val78, respectively, which likely contribute to stability of the inhibitor in the channel (Fig. 2*D*). Further toward the intracellular side of the pore, the ligand forms two hydrogen bonds via its urea NH groups to the carbonyl oxygens of His92 and Ala91, situated on loop B (Fig. 2*E*). Closest to the cytoplasmic side of the pore, the phenyl pyrazole group is accommodated, and the nitrogen of the pyrazole ring hydrogen bonds to the side chain of Gln183, which likely positively affects the specificity and affinity of the compound (Fig. 2*E*). This is supported by that the structurally similar compound 9016645 (Fig. 2*A*), lacking the pyrazole ring, have a significantly lower AQP7 inhibitory capacity (4). CH–π interactions may also contribute to the affinity as Leu186 is facing the aromatic ring of the ligand while the pyrazole ring is positioned toward Phe187 (Fig. 2*D*). In addition, hydrophobic analysis (including residues lining the channel within 5 Å from the inhibitor molecule) reveals that the polar urea is packed toward the hydrophilic side of the channel (His92, Asn94, and Asn226) while the relatively polar pyrazole ring is close to the hydrophilic region formed by His92, Asn101, Arg106, and Gln183. Conversely, the two phenyl groups in the inhibitor are packed against the hydrophobic side of the channel with residues, Val78, Val82, Leu182, Leu186, Ile206, and Val210 (*SI Appendix*, Fig. S5). Thus, the overall amphipathic character of the inhibitor is congruent with that of the glycerol channel.

### Simulations Suggest Distinct Inhibitor Configurations.

**Fig. 3.**
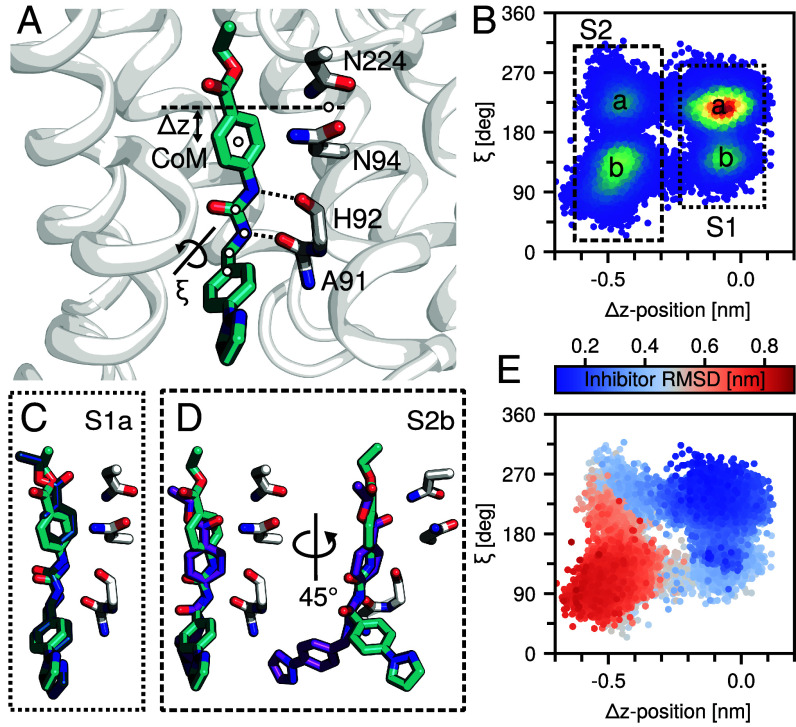
Cluster analysis of AQP7-inhibitor structures. (*A*) Two variables are used to cluster the protein-ligand structures: 1) the center of mass distances between the upper ring and the NPA residues; and 2) a torsion describing the lower ring rotation about the pore axis. (*B*) Sampling density within the 2D space. Two main clusters (S1/S2) and two subclusters in each (a/b) are indicated. (*C* and *D*) Centroids from S1a and S2b and cryo-EM (cyan). S2b is shown at two angles to reveal the torsional rotation of the lower ring. (*E*) Inhibitor RMSD following a backbone and inhibitor alignment. Blue indicates good agreement; red indicates poor. The most frequently sampled cluster (S1a) had the lowest RMSD.

The cryo-EM map displays a clear density with a distinct bend toward the intracellular side of the channel, which allows us to confidently conclude the positioning of the inhibitor. To provide further insight into the dynamics of Z433927330 and the nature of its binding mode and interactions with the channel residues in AQP7, extensive MD simulations were performed. No unbinding of the inhibitor was observed during the simulation; however, movements along the channel axis (i.e., *z*-axis) were observed. Based on principal component analyses (PCA), two key descriptors of the ligand within the pore were resolved: 1) Δz-position, corresponding to the distance between the center of mass (CoM) of the NPA domain and the CoM of the upper ring; and 2) a torsion angle, describing a lower ring rotation (Fig. 3*A*). Projection of the trajectories in these two dimensions resolved four clusters (Fig. 3 *B*–*D*). The two clusters that remained closer to the cryo-EM z-position were identified as S1 and those that shift toward the intracellular opening of AQP7 as S2 (note that a and b are used to denote the ring torsion rotation). Cluster S1a (Fig. 3*C*) was sampled most frequently (~52%) and had the lowest RMSD with the cryo-EM structure (Fig. 3*E*), while cluster S2b (Fig. 3*D*) which was sampled second most frequently (~21%), had the highest RMSD from the cryo-EM owing to a significant ring rotation (Fig. 3*E*). In all structures, hydrogen bonds between the carbonyl oxygens of Ala91 and His92 and the urea NH groups in the ligand were observed. We note that these simulations were performed at room temperature and the significance of the deepest S1a free energy minimum may increase substantially and asymmetrically relative to the other minima at lower temperature. Such an effect would result in ensemble contraction and potentially the resolution of only a single cryo-EM structure. Overall, the results suggest that the ligand can adopt two main configurations characterized by a shift along the pore axis. The S1 clusters are “deeper” in the pore and resemble most closely the cryo-EM structure, while the S2 clusters shift toward the intracellular opening. In both clusters, two subpopulations can be characterized by a ring rotation; however, interactions between the channel with this portion of the inhibitor were less pronounced. Permeation analysis revealed no water permeation when the inhibitor was bound to AQP7. This was also supported by experimental data showing that Z433927330 has a clear inhibitory effect of AQP7 in a proteoliposome assay (*SI Appendix*, Fig. S6).

### Ester Group Flip Forms Key Hydrogen Bond(s).

**Fig. 4.**
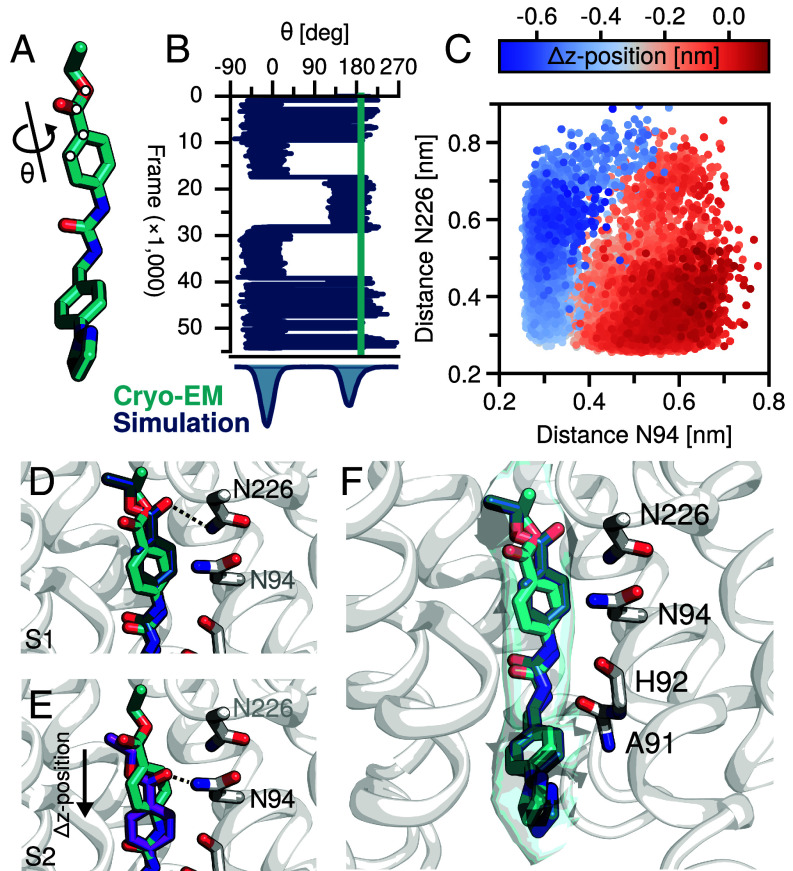
Ethyl-benzoate rotamer and hydrogen bonds. (*A*) Atoms involved in the rotamer. (*B*) Torsional sampling as a function of the number of structures included in the ensemble. Hydrogen bonding is facilitated when the θ ~ 0 rotamer is sampled. (*C*) Heavy atom distance between the ligand ester oxygen and N94/N226 nitrogen. Marker color corresponds to the dz-distance (i.e., red is S1-like; blue is S2-like). (*D*) S1 cluster structure (teal) hydrogen bonding with N226 (*E*) S2 cluster structure (purple) hydrogen bonding with N94; note this is shifted down from cryo-EM (cyan). (*F*) Cryo-EM density, cryo-EM structure, and S1 cluster centroid. Four interacting AQP7 residues are indicated.

In addition to the lower ring dihedral, a second notable dihedral motion was observed, specifically, that involving the ethyl-benzoate moiety of the ligand (Fig. 4*A*). We observed sampling of two torsional configurations, one in agreement with the way the ligand is modeled in the cryo-EM structure and another that facilitates a hydrogen bond with the NPA motif, either Asn94 or Asn226 (Fig. 4*B*). The determinant of whether the bond is with Asn94 or Asn226 depends on the z-position of the ligand (Fig. 4*C*). In the deeper S1 configuration, the hydrogen bond is with Asn226 (Fig. 4*D*), while in the S2 configuration, the hydrogen bond is with Asn94 (Fig. 4*E*). Encouragingly, this torsional rotation appears to be consistent with the cryo-EM density (Fig. 4*F*), suggesting that both rotamers are possible. Given that only one rotamer facilitates the observed NPA-motif hydrogen bond, it seems plausible that this simulation-implied rotamer is more likely.

### Structural Comparison with AQP7_g_.

**Fig. 5.**
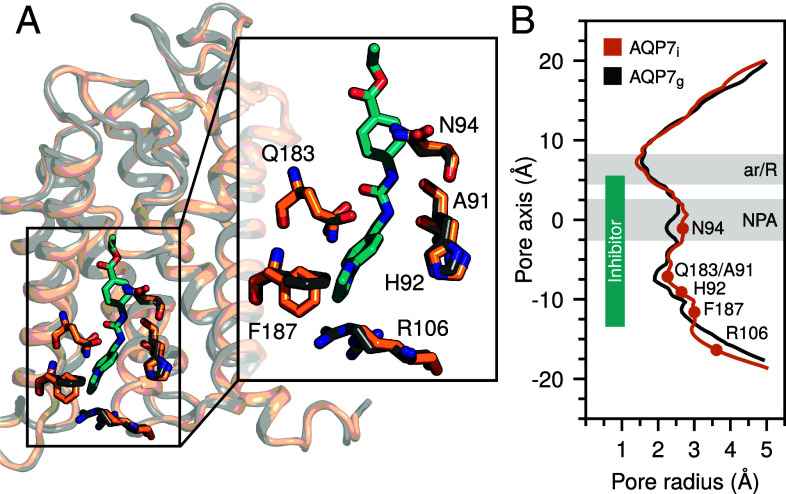
Structural comparison of inhibitor bound (AQP7_i_) and glycerol bound (AQP7_g_) AQP7. (*A*) Structural alignment of AQP7_i_ in deep orange and AQP7_g_ in gray, shown as ribbon. The region boxed is shown as zoom-in and residues are displayed as sticks. (*B*) The radii along the channel (Å) were plotted for AQP7_i_ (inhibitor removed) and AQP7_g_ (glycerols removed). Key residues are presented as dots in the plot, while the relative inhibitor z-position is indicated by a cyan rectangle.

To structurally analyze the molecular consequences on AQP7 upon binding of the inhibitor, the AQP7_i_ cryo-EM model was superimposed with the AQP7_g_ cryo-EM model (PDB ID: 8AMX) (Fig. 5*A*) with a calculated RMSD of 0.3 Å and calculations of the channel widths were performed using HOLE software (Fig. 5*B*). The major residue movements upon inhibitor binding are on the intracellular side. In particular, two residues located at the intracellular vestibule of AQP7_i_, Arg106, and Phe187, display significant shifts in comparison with the AQP7_g_ structure (Fig. 5*A*), resulting in a wider channel at the intracellular side to accommodate the inhibitor (Fig. 5*B*). The Phe187 side chain rotates approximately 90° to avoid steric clashes with the inhibitor and instead it forms a CH–π interaction between its phenyl ring and the carbon atom of the pyrazole ring of the inhibitor (Fig. 5*A*). In addition, we observe that coordinating residues, Ala91, His92, and Gln183, as well as Asn94, show small movements leading to a wider channel upon inhibitor binding (Fig. 5*B*). This correlates well with previously observed dynamic properties of the AQP7 glycerol channel (5).

### The Selectivity of the Z433927330 Inhibitor.

**Fig. 6.**
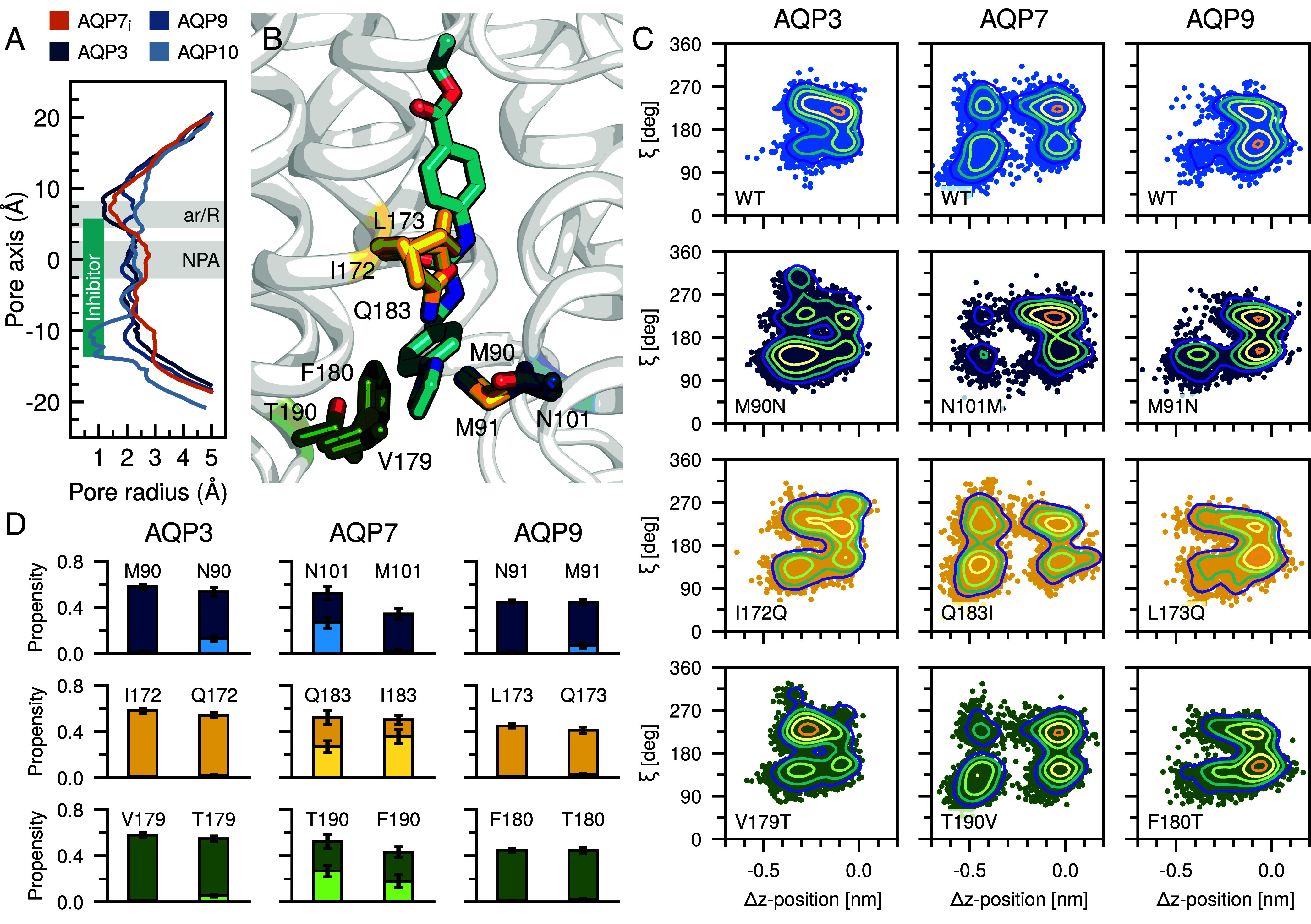
Structural comparison of AQP7_i_ with other human AQPs. (*A*) The radii along channels (Å) were plotted for AQP7_i_ and aquaglyceroporins (AQP3, AQP9 and AQP10). The relative inhibitor z-position along the pore axis is indicated. (*B*) Alignment of AQP7 with AlphaFold2 AQP3 and AQP9 structures (only the specific residues in these structures are shown). (*C*) Projection of trajectories along the Δz-position and torsion angle. (*D*) Interaction propensity for the inhibitor with the upper (dark color) or lower (light color) NPA-motif in the WT and mutant AQPs. SEs are indicated.

In order to resolve the basis for the selectivity of Z433927330 for AQP7, the structure of AQP7_i_ was compared to published structures of human AQPs, i.e., AQP1 (PDB ID:1FQY) (6), AQP2 (PDB ID:4NEF) (7), AQP4 (PDB ID:3GD8) (8), AQP5 (PDB ID:3D9S) (9), AQP10 (PDB ID:6F7H) (10). From HOLE analyses, it is clear that all the orthodox AQPs (AQP1, AQP2, AQP4, AQP5) have significantly narrower channels than AQP7 (*SI Appendix*, Fig. S7), suggesting that the rather bulky Z433927330 inhibitor cannot fit into the channels of these AQPs. Similarly, AQP10, an aquaglyceroporin, has a closed configuration from the endofacial side in the structure (Fig. 6*A*) (10) and consequently binding of the inhibitor is blocked. Previous work suggests Z433927330 has an inhibitory capacity five times lower for AQP3 and AQP9 than for AQP7 (4). Alignment of AQP7 structure with AlphaFold2 models of AQP3 and AQP9 suggested three residue sites (corresponding to Asn101, Gln183, and Thr190 in AQP7) that may play a role in affecting inhibitor binding within the channel (Fig. 6*B*). To investigate this, MD simulations of inhibitor bound AQP3 and AQP9 AlphaFold2 structures were executed. Projecting our trajectories along the same two descriptors described previously (i.e., Δz-position and torsion angle), we observed that unlike in AQP7, the inhibitor less frequently sampled configurations toward the intracellular opening in AQP3 and AQP9 (Fig. 6*C*, upper row). Notably, a methionine that is conserved in both AQP3 (Met90) and AQP9 (Met91) is replaced with an asparagine (Asn101) in AQP7 (Fig. 6*B*). To resolve the role of the methionines, these were substituted to asparagines in AQP3 and AQP9 and vice versa in AQP7, to make AQP7 more like AQP3 and AQP9. The Met90Asn and Met91Asn mutations in AQP3 and AQP9, respectively, resulted in more frequent sampling of lower conformations and a significant increase in the number of interactions with the lower motif (Fig. 6*C*, second row). Conversely, the Asn101Met mutation in AQP7 resulted in a significant reduction in sampling within this region and a reduction in the number of interactions with the lower motif (Fig. 6*C*, second row). A qualitatively similar reduction in the lower NPA-motif interactions for the Thr190Phe/Val mutant in AQP7 is detected, however, this is less quantitatively pronounced than in the Met90/Met91/Asn101 case (Fig. 6*D*). Similarly, substitution of the third site (Gln183) was also less quantitatively pronounced. In all cases, when substitutions are introduced into AQP3 and AQP9, the constricted sampling of Z433927330 is in part reduced, allowing for a larger sampling of the binding site and in some cases the facilitation of new interactions. It should be noted that here we have only considered bound state structures; thus, the results do not preclude the importance of other residues for facilitating the initial binding. Overall, the findings suggest that inhibitor interactions with the upper and lower motifs may contribute to altered affinities: an increased affinity, as in the case of AQP7 where both interactions are engaged, and a decreased affinity in AQP3 and AQP9 where only interactions with the upper motif occur.

### The Z433927330 Compound Inhibits Cancer Cell Proliferation.

**Fig. 7.**
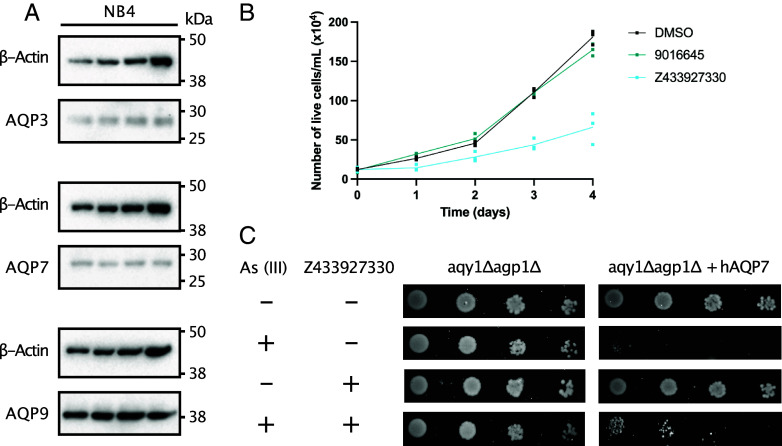
Compound Z433927330 inhibits human AQP7 activity and reduces cancer cell proliferation. (*A*) AQP3, AQP7 and AQP9 expression in the NB4 cell line (four replicates). β-Actin was used as loading a control. (*B*) Number of live cells/mL analyzed by trypan exclusion assay over 4 d. Cells were monitored by counting of live NB4 cells after addition of 10 µM Z433927330 or 9016645 or DMSO as control, n = 3. (*C*) Yeast growth assay in the presence of either 300 µM As(III) and/or 100 µM Z433927330. The assay was repeated at least two times independently with same result.

Recently it was reported that AQP7 is a vulnerability in cancer (3). Thus, inhibitors of AQPs with translational potential are relevant. Since diminished expression of AQP7 in a mouse breast cancer model caused reduced primary tumor burden (3), and cell proliferation in leukemia cells is dependent on intracellular glycerol levels (11), we investigated the translational properties of Z433927330. Cell proliferation was analyzed in the acute promyelocytic leukemia (APL) cell line NB4 which showed expression of all three aquaglyceroporins AQP3, AQP7, and AQP9 (Fig. 7*A* and *SI Appendix*, Fig. S8). The variation in band intensity and apparent molecular weight of AQPs shown on the membrane is likely due to the use of protein-specific antibodies and potential differences in glycosylation levels. Z433927330 is known to be an efficacious inhibitor of AQP7 activity (IC_50_~0.2 µM) while inhibition of AQP3 and AQP9 is lower (IC_50_~ 1 µM) (4). On the other hand, the 9016645 compound (lacking the pyrazole ring but otherwise identical to Z433927330) (Fig. 2*A*) is reported to have no inhibitory activity for AQP7 (4). Upon treatment with Z433927330, cell proliferation was clearly affected leading to a reduced number of live cells over time, while no effect was detected for 9016645 (Fig. 7*B*, and *SI Appendix*, Fig. S9). This is in agreement with the previously reported structure–activity relationship for these inhibitors (4).

To confirm that the reported AQP7 targeting compound Z433927330 inhibits the activity of human AQP7, a yeast growth assay was set up. A *Pichia pastoris* strain deficient in endogenous AQPs, Aqy1 and Agp1 proteins, was transfected with human AQP7 (12). AQP7 is known to facilitate the transport of arsenic trioxide (As(III)) and the accumulation of As(III) is toxic to yeast (12). As evident in Fig. 7*C*, *P. pastoris* double-deletion strain (Δ*aqy1*Δ*agp1*) cells expressing AQP7 could not survive at high concentration of As(III), while those not expressing AQP7 could survive (Fig. 7*C*). This indicates that AQP7 is an efficient As(III) facilitator and is in agreement with previous findings (12). To investigate whether arsenic trioxide uptake through human AQP7 can be inhibited by compound Z433927330, cells were grown on plates including both As(III) and Z433927330. Consistent with previously published mouse AQP7 data, cells expressing human AQP7 are rescued in the presence of Z433927330, confirming that the inhibitor targets human AQP7 and blocks the uptake of As(III) (Fig. 7*C*). Although aquaglyceroporins are the major facilitators of arsenic trioxide in yeast, As(III) can also pass through the hexose transporters (13); this could explain why Z433927330 does not completely rescue the cells.

## Discussion

Owing to their fundamental role in maintaining cellular homeostasis, AQPs have been rigorously investigated since their discovery in 1991 (14). The elevated expression of AQP isoforms in cancer has opened the possibility of exploiting AQP inhibitors for cancer treatment. Recently, AQP7 was identified as a novel regulator of breast cancer and reduced expression resulted in decreased primary tumor burden and lung metastasis (3). This finding suggests that AQP7 is a cancer-specific vulnerability. To date, several small molecules and heavy metal compounds have been reported to block the activity of AQPs (2); however, the drug discovery process has been severely hampered by the lack of three-dimensional structures of AQPs in complex with inhibitors. Here, we report structural analyses of AQP7 in complex with the inhibitory compound Z433927330 and a proliferation assay showing a reduction in proliferation of cancer cells treated with Z433927330. We expect that revealing the structural and molecular basis for inhibition of AQP activity has the potential to facilitate future development of AQP isoform-selective inhibitors for use as cancer therapeutics.

Finding inhibitors specific for AQP isoforms has been considerably more challenging than expected due to the high level of structural conservation within the AQP family. The availability of an AQP structure in complex with an inhibitor allows for a detailed structural isoform comparison and provides a necessary template to guide the design of an AQP7-specific inhibitor. It also serves as a key starting point for further in silico investigation. One possibility would be to use relative binding free energy calculations—based on non-equilibrium switching (15) or free energy perturbation (16)—to resolve the effects of ligand modification (15) or protein substitutions on binding affinity (17). A second possibility would be to further investigate how those residues near the cytoplasmic opening (e.g., Asn101, Gln183, and Thr190) impact inhibitor binding. We have performed simulations starting from the bound state structure; however, the experimentally observed selectivity of Z433927330 for AQP7 compared to AQP3 and AQP9 may be more clearly resolved by considering how the inhibitor binds to these channels using a method like funnel metadynamics (18).

The AQP7_i_ cryo-EM model was compared to the related AQP3 and AQP9 models, as Z433927330 is reported to bind these other AQPs, but have lower potency (4). The structural findings in combination with MD simulations suggest that the inhibitor can adopt two main configurations characterized by a shift along the pore axis, with the upper configuration more prominent. The shift along the axis likely contributes to an increased affinity for AQP7, compared to AQP3 and AQP9 where only one configuration is adopted. Thus, refinement or extension of the ligand scaffold could be a valid approach to further develop the compound and improve affinity. In the study by Sonntag et al. describing the discovery of Z433927330 as an AQP7 inhibitor, it was noted that the aromatic/arginine region of the pore is too narrow to contain inhibitors. However, the crystal structure of AQP7 (19) as well as the previously published cryo-EM structures with glycerol (5), reveal a glycerol molecule in the aromatic/arginine filter, binding to the side chain of Arg229 and to the main chain carbonyls. In light of this, the possibility to extend the compound toward the extracellular side of the aromatic/arginine filter (e.g., by modifying the ethyl benzoate moiety to a glyceryl benzoate), should be explored. The pyrazole ring is also an important moiety for maintaining the affinity of the compound, as cell proliferation assays showed no inhibitory effect with a related compound lacking this moiety. Regarding specific ligand–protein interactions, three amino acid residues at the cytoplasmic opening of the channel stand out as good targets to tailor isoform-selective AQP7 inhibitors (Asn101, Gln183, and Thr190) as these correspond to methionines, isoleucine/leucin, and valine/phenylalanine in AQP3 and AQP9, respectively. Introducing additional moieties capable of participating in hydrogen bonds with Asn101 and Thr190 might not only improve the affinity of the compound but also make it selective toward AQP7 as bulkier residues in both AQP3 and AQP9 would not allow for the accommodation of extra functional groups in this area. Additional hydroxyl or amino moieties could also be introduced that would potentially hydrogen bond Thr190 and/or Asn101. Taken together, the experimental structure of AQP7 in complex with an inhibitor compound presented here provides a necessary blueprint for further compound development and drug discovery within the field of AQP research.

## Material and Methods

### AQP7 with Inhibitor Sample Preparation for cryo-EM.

Human AQP7 was expressed and purified as described previously (20). Briefly, AQP7 was over-expressed in *P. pastoris* before the membranes were isolated and solubilized in 1 % n-decyl β-D-maltopyranoside (DM, Anatrace), subsequently, the sample was further purified in glycol-diosgenin (GDN, Anatrace) by affinity chromatography (Ni-NTA resin, Invitrogen) and size exclusion chromatography (Superose 6 Increase 10/300 GL, Cytiva) for cryo-EM grid preparation. The inhibitor (Z433927330, Axon Medchem) in final concentration of 25 µM was mixed with 1 mg/mL AQP7 protein sample prior to plunge-freezing on the cryo-EM grids (C-flat Cu R1.2/1.3, 300 mesh) using a Vitrobot MarK IV (FEI) at 4 °C and 100 % humidity. Grids were examined on a Titan Krios electron microscope (FEI) operating at 300 kV equipped with a spherical aberration (Cs) image corrector. Micrographs were recorded using a K3 Summit direct electron detector in super-resolution mode under the 105,000× magnification. Forty dose fractions were exposed for 2.2 s, and thus, the total dose for each stack was ∼49.448 e−/Å^2^. All 40 dose fractions were aligned, summed, dose weighted, and twofold binned to a pixel size of 0.8464 Å. Finally, approximately 9,000 micrographs were collected using the parameters above.

All collected micrographs were motion-corrected, and contrast transfer function (CTF) was estimated in cryoSPARC (21); then, 4,239 micrographs were left in the dataset for the further particle picking after manual inspection to discard poor micrographs. Next, the AQP7 cryo-EM map (EMDB ID: EMD-15527) was imported to generate a series of projections to be used as templates for particle picking from the above micrographs. The picked particles were extracted and classified by 2D classification, from which five classes were identified by applying ab initio reconstruction and then subjected to heterogeneous refinement. Finally, the particles and the volume presenting the octameric class were selected and further refined by non-uniform refinement using D4 symmetry and CTF refinement. This generated the cryo-EM map at an overall resolution of 3.2 Å as estimated by the gold-standard Fourier shell correlation with a cutoff at 0.143.

### Model Building and Refinement.

The AQP7 cryo-EM model (PDB ID:8AMW) was fitted into the cryo-EM map in Chimera (22), after which the model was refined by real space refinement in Phenix (23) and edited manually using coot (24). The inhibitor structure was built and optimized using eLBOW, a built-in tool in Phenix, and then fitted into the cryo-EM map before the real space refinement in Phenix (23). Finally, the model of the protein–inhibitor complex was subjected to a real space refinement in Phenix for the statistics calculations.

### HOLE Analysis of Channels.

HOLE software was downloaded from http://www.holeprogram.org/ (25), and the dimension of water/glycerol channels was calculated with an end radius of 5 Å. The inhibitor and glycerol are removed from coordinates of inhibitor bound (AQP7i) and glycerol-bound (AQP7g) AQP7 before HOLE calculation, respectively. All pore profiles were visualized by Pymol (version 2.3.4).

### MD Simulations.

All simulations were performed using GROMACS (26) software and the CHARMM36m force field (27). Z433927330 was parameterized using https://cgenff.paramchem.org (28, 29). Initial parameter quality was exceptionally high (i.e., all penalties less than 5), and no optimization was performed. Individual system configurations were prepared using Charmm-GUI (30) embedding the wild-type AQP3, AQP7, and AQP9 tetramers into lipid bilayer patches of 168 POPC lipids (31). Boxes were solvated with CHARMM TIP3P water molecules (32), and sufficient potassium and chloride atoms were added to ensure charge neutrality and to reach an ionic strength of 150 mM. Mutations were introduced into the wild-type structural configurations. Initial AQP3 (AF-Q92482) and AQP9 (AF-O43315) were taken from the AlphaFold2-EMBL database (33, 34). All systems were subject to an initial energy minimization using the steepest descents algorithm. The temperature was maintained at 300 K using the Parrinello–Donadio–Bussi velocity rescaling method (35) with a coupling time of 1.0 ps, while the pressure was maintained at 1 bar using the C-rescale barostat (36) with a coupling time constant of 5.0 ps. The simulation time step was 2.0 fs. Long-range electrostatic interactions were calculated using the particle–mesh Ewald method (37) with a Fourier spacing of 0.12 nm and a real-space cutoff of 1.0 nm. Lennard–Jones interactions were force-switched off between 1 and 1.2 nm. Bond lengths were constrained using the Parallel LINear Constraint Solver (38). For each system, five production simulations of between 1.5 and 3.0 μs were performed. The first 100 ns was discarded as equilibration. For analysis purposes, monomers were assumed to be independent.

### Proteoliposome Assay.

The proteoliposome assay was carried out as the STAR protocol by Steffen et al. (https://doi.org/10.1016/j.xpro.2022.101312). Briefly, *Escherichia coli* polar lipid extracts were prepared by dehydration to create a lipid film of preformed liposomes (20 mg/mL). First, 700 µL of the solution was sonicated in glass vials for 3 × 15 min. The lipids were flash-frozen in liquid nitrogen and left to thaw at RT. Then, this freeze-and-thaw step was repeated three times. Support filters and 0.2 µm filters were equilibrated using reconstitution buffer (20 mM Tris pH 7.5, 200 mM NaCl, 25 % glycerol). The lipids were passed through the extruder (Mini-Extruder, Avanti) 11 times and diluted to a concentration of 4 mg/mL in a reconstitution buffer containing 0.1 % DM. Then, 40 µL Triton X-100 was added to the sample to a final concentration of 1.6 %. AQP7 was added to the lipid solution in a lipid-to-protein ratio (LPR) of 50 and incubated for 1 h at 4 °C. The samples were dialyzed O/N against 1 L reconstitution buffer at 4 °C to remove detergent and residual external fluorophore. The next day, samples were centrifuged at 23,000 rpm for 1.5 h in the Sorvall Lynx 6000 cell centrifuge (Thermo Fisher). Pellets were resuspended in 1 mL resuspension buffer (20 mM Tris pH 7.5, 200 mM NaCl). To asses inhibition of AQP7 activity, 25 µM Z433927330 was incubated with a LPR50 sample for 1 h on ice.

### Water Permeability Measurements.

Stopped-flow experiments on proteoliposomes and empty liposomes were performed on an SX-20 Stopped-flow Spectrometer system (Applied Photophysics). Empty liposomes were used as a negative control. The proteoliposomes were rapidly mixed with the reaction buffer (20 mM Tris pH 7.5, 200 mM NaCl, and 570 mM sucrose) and measured for fast kinetics. Data were collected at 20 °C for 2 s at 495 nm. The data were analyzed and plotted using GraphPad Prism (v. 9.5.1). Each sample was averaged from 10 to 15 readings from three independent proteoliposome preparation batches, normalized, and fitted to a double exponential equation.

### Human Cell Culture.

NB4 cells were obtained from the German Collection of Microorganisms and Cell Cultures, (Braunschweig, Germany), and maintained in RPMI 1640 medium (Thermo Fisher Scientific) supplemented with 10 % FBS and 1 % penicillin/streptomycin at 37 °C and 5 % CO. The day before the start of experiments, cells were split in 2/3 fresh media and monitored for proper cell viability.

### Western Blotting.

NB4 cells were seeded into four separate wells in a 12-well plate with 300,000 cells/well. After 72 h, the cells were spun down and pellets resuspended in lysis buffer consisting of Tris-HCl (50 mM, pH 7.4), sodium pyrophosphate (5 mM), NaF (50 mM), EDTA (1 mM), EGTA (1 mM), sucrose (270 mM), DTT (1 mM), sodium orthovanadate (50 μM), NP-40 (1 % w/v), and cOmplete™ protease inhibitor cocktail (Merck). Lysates were centrifuged at 14,000*g* (15 min, 4 °C). The protein concentration was determined by Bicinchoninic Acid Protein analysis (Merck), and 25 μg was loaded to an SDS-PAGE gel with each of the four samples in triplicates. After electrophoresis, the protein bands were transferred to a nitrocellulose membrane with wet transfer followed by 1 h blocking with skim milk (10 % w/v) in TBS (0.1 % Tween20). The membrane was cut and incubated over night at 4 °C with primary antibodies against AQP3 (1/1,000, Abcam ab125219), AQP7 (1/500, Abcam ab32826), or AQP9 (1/500, Santa Cruz Biotechnology sc-14988) diluted in TBS (0.1 % Tween20) with BSA (5 % w/v). After washing, blots were incubated (2 h, 4 °C) with secondary antibodies, diluted in TBS (0.1 % Tween20) with skim milk (5 % w/v), recognizing rabbit (1/5,000, Invitrogen 31460) or goat (1/1,000, R&D Systems HAF019). Blots were developed using Chemiluminescent Substrate (West Pico PLUS, Thermo Scientific). For loading control, the membrane was incubated with Restore^TM^ PLUS Western Blot Stripping Buffer (Thermo Scientific) for 10 min and re-blocked and re-probed with primary anti-β-actin (1/1,000, Abcam ab8227) and secondary anti-rabbit (1/5,000, Invitrogen 31460) antibodies.

### Trypan Blue Proliferation Assay.

NB4 cells were seeded into a 96-well plate with 15,000 cells per well. Compounds Z433927330 (Axon Medchem) and 9016645 (ChemBridge Corporation) were dissolved in DMSO to 1,000× solutions and diluted several times in cell culture media before adding to cells in concentrations of 5 and 10 μM. DMSO was used as control. All treatments were added to three wells separately. The final concentration of DMSO in the samples was 0.1 %. Live cells were counted using trypan blue directly after seeding and live cell counts were then monitored for each well during the following 4 d. The samples were counted at the same time each day and treated in the same way by mixing with a pipette and removing 10 μL of cells to be mixed with 10 μL of trypan blue. Live cells/mL were analyzed with an automatic cell counter (Countess II FL, Thermo Fisher).

### Yeast Growth Assay.

The assay was performed as described previously (12) with a modification of including the inhibitor Z433927330 in the medium. Briefly, the double-deletion GS115 strain (*aqy1Δagp1Δ*), was transfected with the human AQP7 gene (12). The double-deletion strain and the strain expressing human AQP7 were cultured, and protein expression was induced in methanol-containing media (39). After 6 h expression, cells were harvested and diluted into 20 mM HEPES pH 7.5 to reach an absorbance (A_600_) of 0.2. Five microliters of cells from 10-time dilution series in HEPES buffer were dropped on BMMY agar plates (1 % w/v yeast extract, 2 % w/v peptone, 100 mM potassium phosphate pH 6.0, 1.34 % w/v yeast nitrogen base, 0.4 mg/L biotin, 0.5 % v/v methanol, 2 % w/v agar) prepared beforehand by supplementing with either NaAsO_2_ (Sigma) and/or the inhibitor. Plates were incubated at 30 °C and growth was monitored over 4 d. The assay has been repeated independently at least two times with the same result.

## Supplementary Material

Appendix 01 (PDF)

## Data Availability

The model coordinates for D4 symmetry applied 3.2-Å structure have been deposited in the Protein Data Bank with the accession code PDB ID 8C9H (40). The corresponding cryo-EM map has been deposited in the Electron Microscopy Data Bank with the accession code EMD-16510 (41). Other relevant data are available from the corresponding author upon reasonable request.
