# Supplementary material for: Molecular basis for human aquaporin inhibition
Source: Proc Natl Acad Sci U S A. 2024 Feb 6;121(7):e2319682121. doi: 10.1073/pnas.2319682121 (PMC10873552; doi:10.1073/pnas.2319682121)
Supplement: Supplementary file 1 — Appendix 01 (PDF) [file pnas.2319682121.sapp.pdf]

Extended Data for

Molecular basis for human aquaporin inhibition

Peng Huang[1*], Hannah Åbacka[1], Carter J. Wilson[2], Malene Lykke Wind[3], Michael Rűtzler[4,5], Anna Hagström-Andersson[6], Pontus Gourdon[1, 3], Bert L. de Groot[2], Raminta Venskutonytė [1, 7,#], Karin Lindkvist-Petersson[1, 7,#*]

1. Department of Experimental Medical Science, Lund University, Lund, Sweden.
2. Computational Biomolecular Dynamics Group, Department of Theoretical and Computational Biophysics, Max Planck Institute for Multidisciplinary Sciences, 37077 Gottingen, Germany
3. Department of Biomedical Sciences, Copenhagen University, Maersk Tower 7-9, Nørre Allé 14, DK-2200, Copenhagen N, Denmark
4. ApoGlyx AB, Lund, Sweden.
5. Division of Biochemistry and Structural Biology, Department of Chemistry, Lund University, Lund, Sweden.
6. Department of Laboratory Medicine, Division of Clinical Genetics, Lund University, Lund, Sweden.
7. LINXS - Lund Institute of Advanced Neutron and X-ray Science, Lund, Sweden.

[*]To whom correspondence may be addressed: Dr. Karin Lindkvist, Department of Experimental Medical Science, Lund University, BMC C13, 221 84 Lund, Sweden, +46 46 2228041, E-mail: karin.lindkvist@med.lu.se or peng.huang@stanford.edu

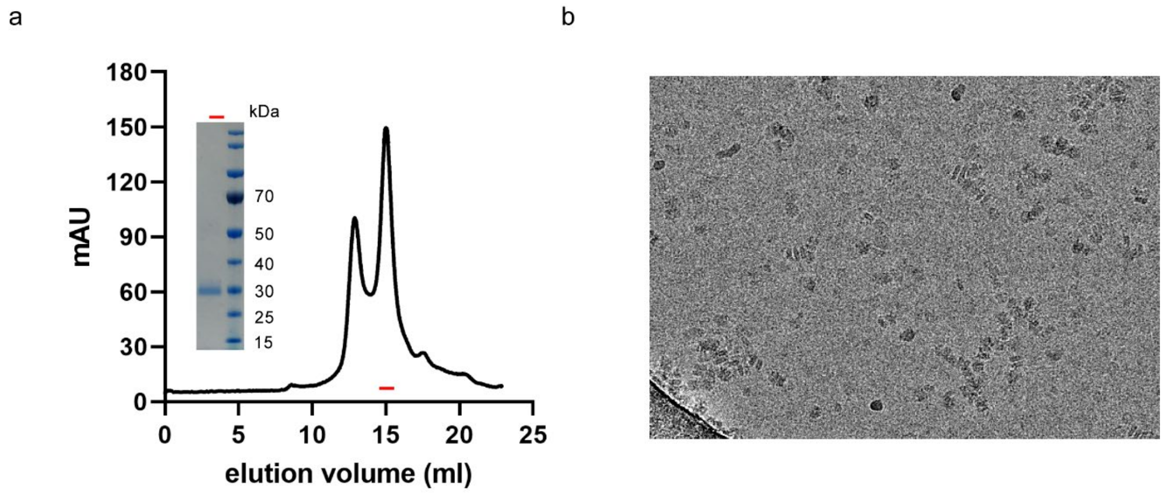

Extended Data Fig. 1 Cryo-EM sample and grid preparation. (a) Size exclusion chromatographic profile and Coomassie-stained SDS-PAGE for purified AQP7. The fractions used for grid preparation were labeled by red line. (b) AQP7 particles distribution on cryo-EM grid.

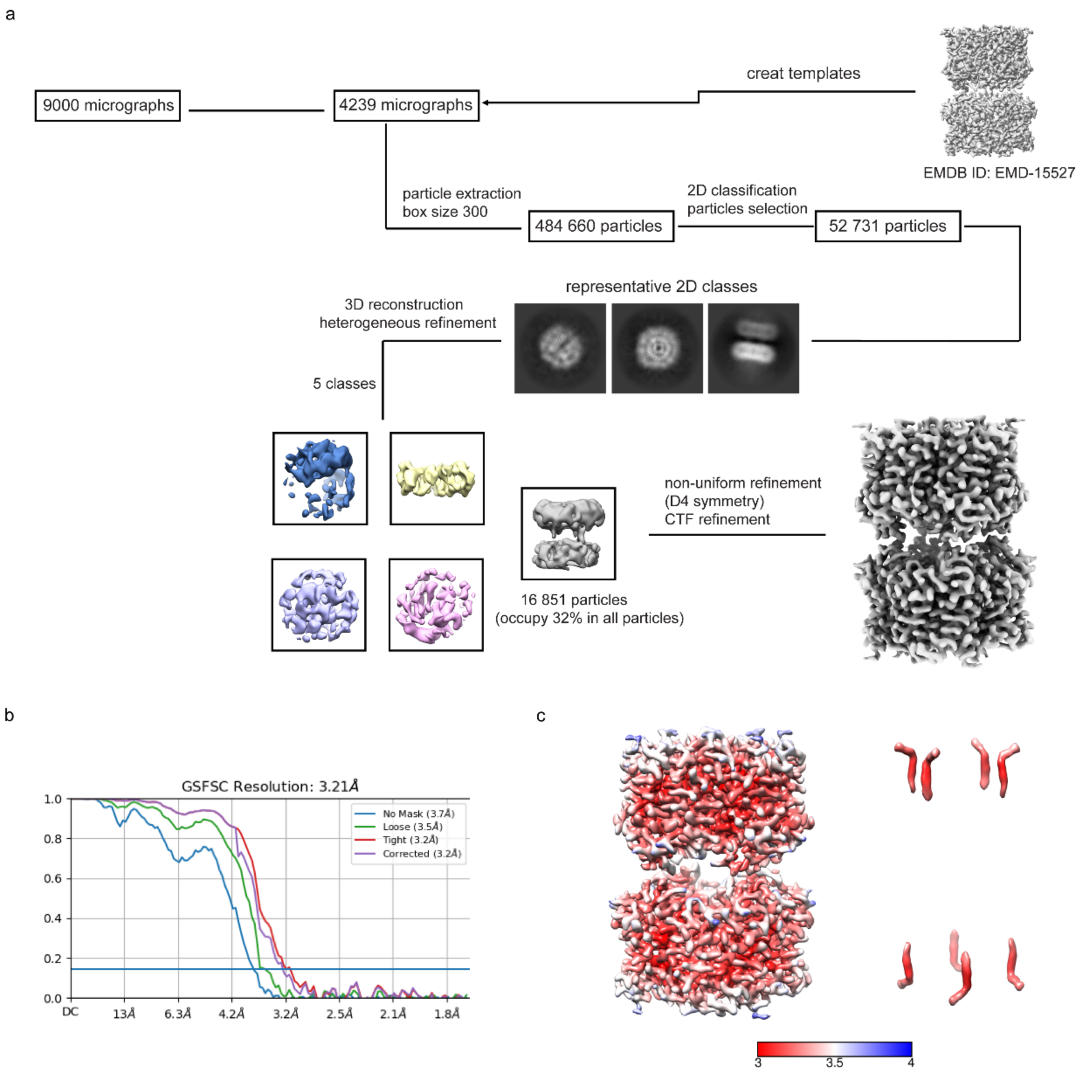

Extended Data Fig. 2 Cryo-EM data processing by cryoSPARC. (a) Data processing pipeline. (b) Global resolution analysis for the overall map in the final refinement. (c) Local resolution analysis for the overall map (left) and inhibitor map (right) in the final refinement.

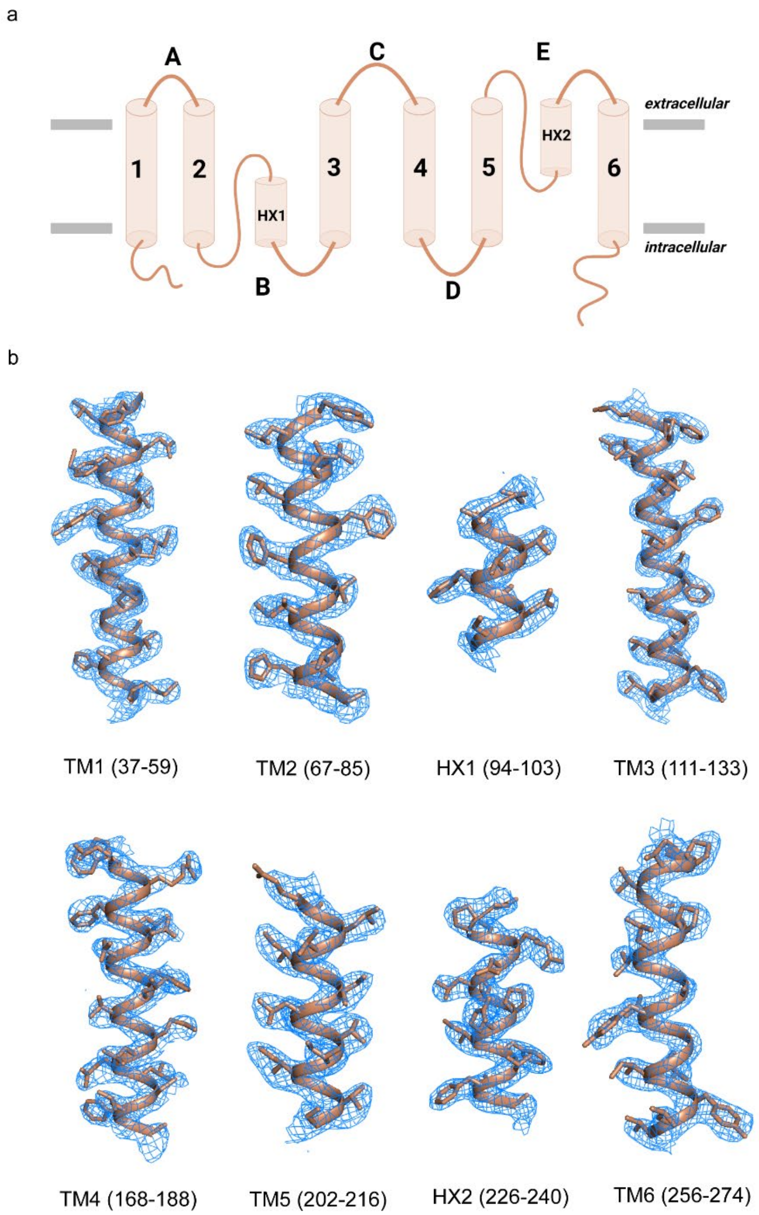

Extended Data Fig. 3 Structural features of AQP7. (a) Topological representation of AQP7, and (b) cryo-EM map for representative fragments in $AQP7_i$.

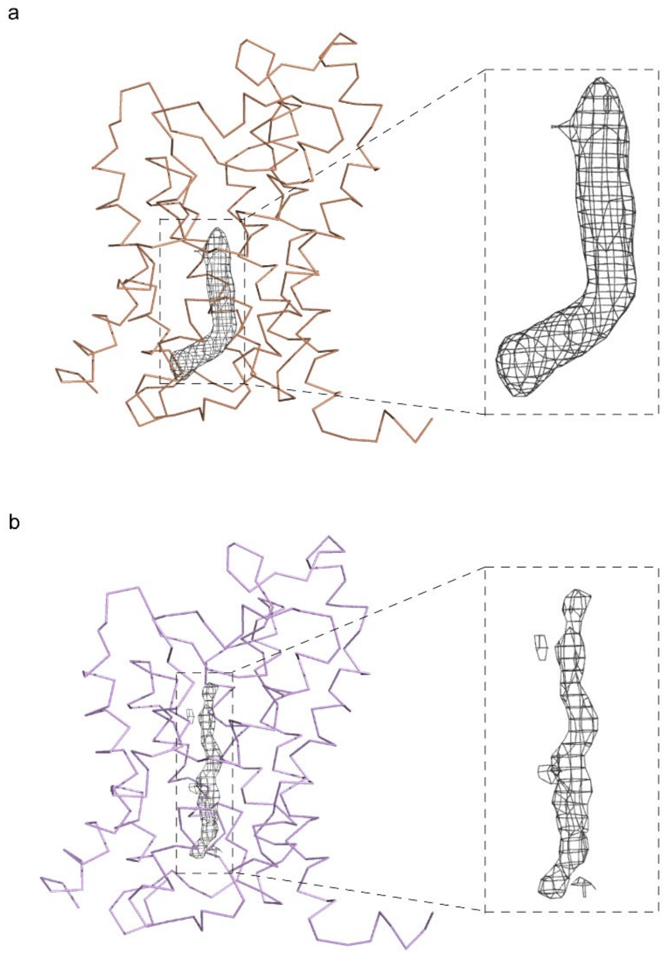

Extended Data Fig. 4 Cryo-EM density comparison for inhibitor in $AQP7_i$ model (a) and glycerols in $AQP7_g$ model. Two densities are shown in gray mesh in same contour level.

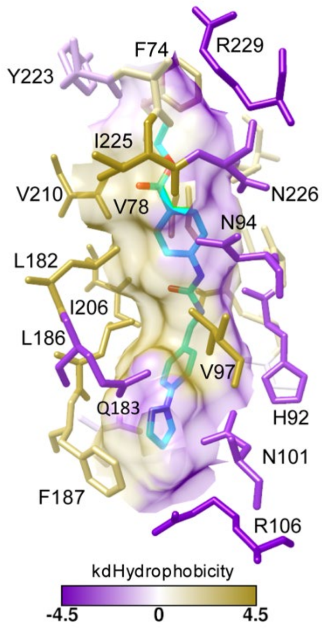

Extended data Fig 5. Hydrophobicity analysis of AQP7 channel. All residues within 5 Å are shown as sticks and colored by the hydrophobicity scale.

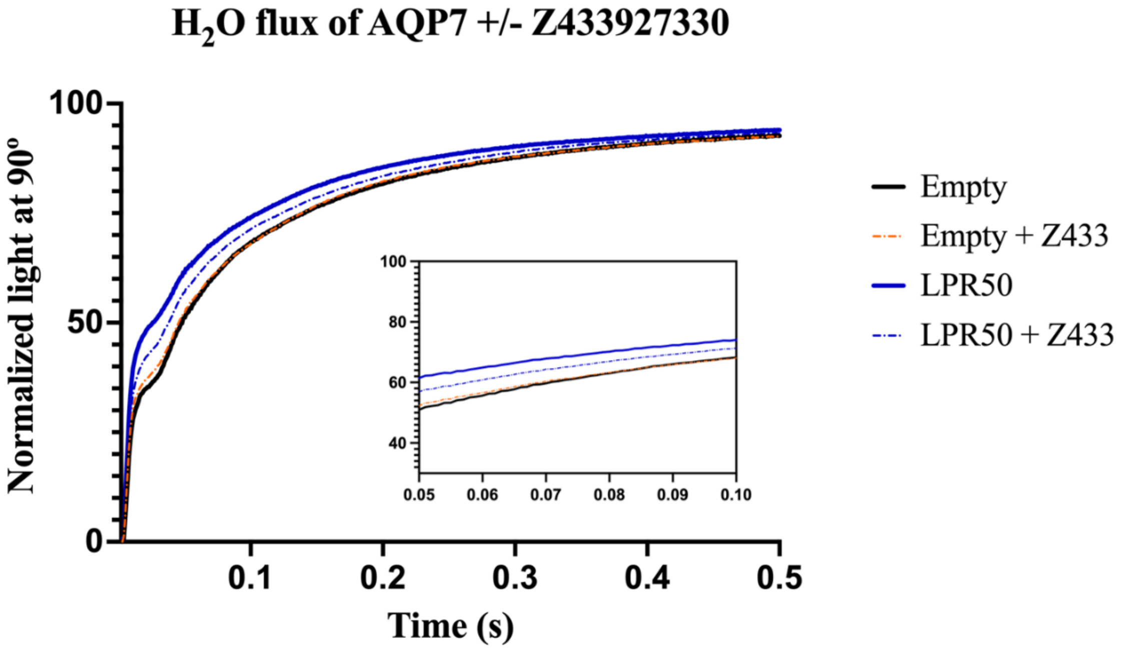

Extended Data Fig. 6. Proteoliposome assay showing H_2O flux for empty liposomes (black trace) and AQP7-embedded proteoliposomes (blue trace) in the absence or presence of 25 µM Z433927330 measured by stopped-flow spectrometry (stippled traces). Trace lines are normalized and fit to a two-phase exponential decay equation. Inset shows zoom in on graph from 0.05 s to 0.1 s.

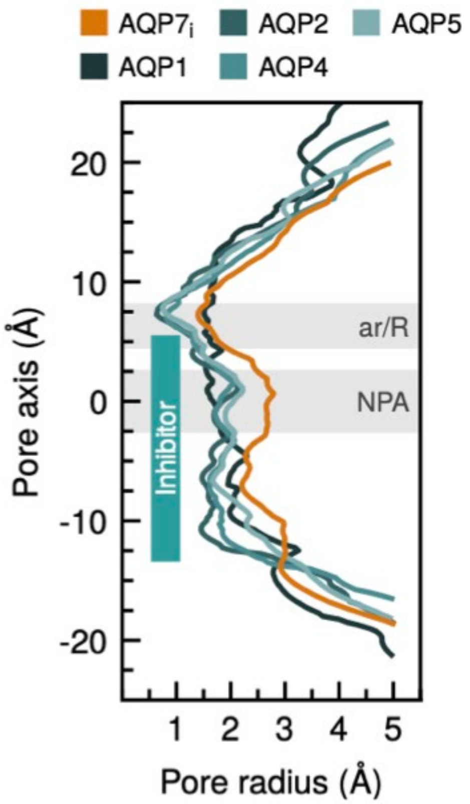

Extended Data Fig 7. HOLE analysis comparison to orthodox AQPs. The radii along channels (Å) were plotted for $AQP7_i$ and orthodox AQPs (AQP1, AQP2, AQP4 and AQP5).

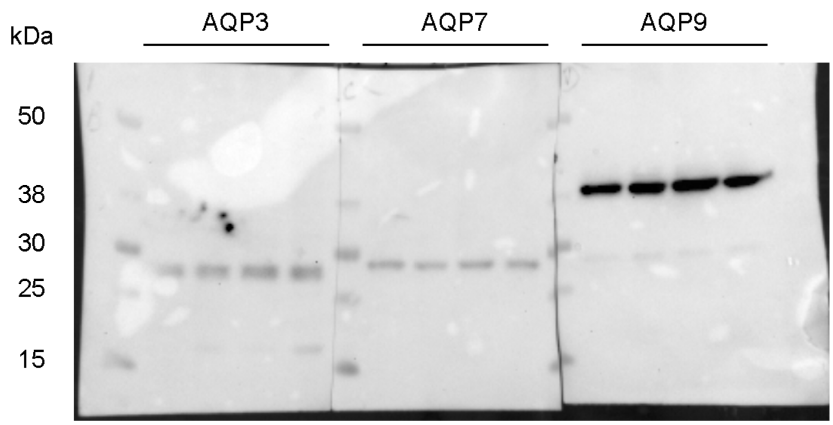

Extended Data Fig. 8 Western blot of AQP3, AQP7 and AQP9 whole lane bands superimposed with picture showing the protein ladder. Lysates are from acute promyelocytic leukemia NB4 cells, n = 4.

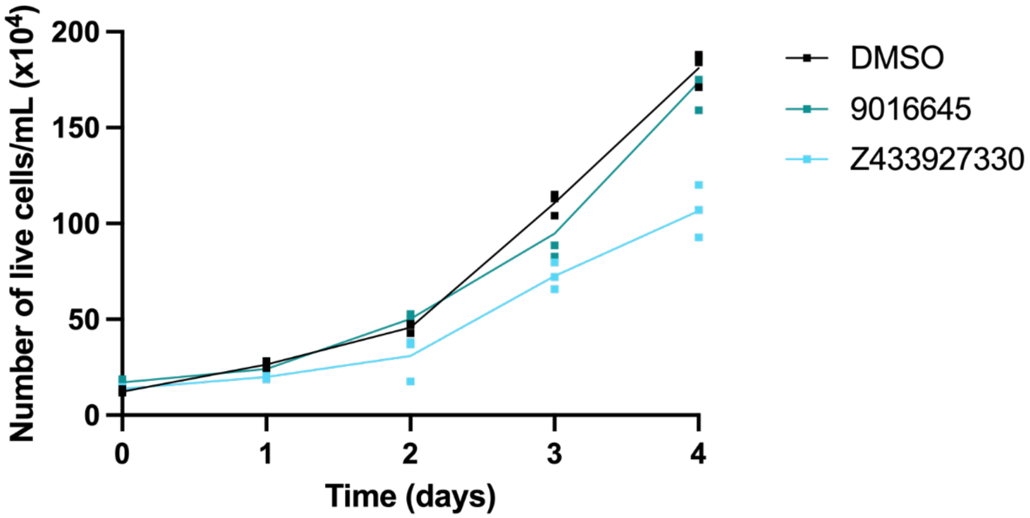

Extended Data Fig. 9 Trypan blue analysis of the number of live cells/mL over four days. NB4 cells were calculated for four days after treatment with 5 μM Z433927330 or 9016645 or DMSO as control, n = 3.

Extended Data Table 1. Cryo-EM data collection, refinement and model statistics for $AQP7_i$

**Data collection and processing**	
Microscopy	FEI Titan Krios
Voltage (kV)	300
Total exposure dose (e^{-}/ Å^2)	50
Frame	40
Pixel size (Å)	0.8464
Data process software	cryoSPARC
Symmetry imposed	D4
Map resolution (Å)	3.2
FSC threshold	0.143
**Refinement**	
Map sharpening *B* factor (Å^2)	108
Refinement software	PHENIX
Refinement tool	Real space refinement
Model composition	
Non-hydrogen atoms	15776
Protein residues	2024
Ligands	LIG:8
B factors (Å^2)	
Protein	45.45
Ligand	28.18
R.m.s. deviations	
Bond lengths (Å)	0.002
Bond angles (°)	0.486
Validation	
Molprobity score	1.52
Clash score	4.90
Rotamers outlier (%)	0.44
CaBLAM outlier (%)	2.81
Cß outlier (%)	0
Ramachandran plot	
Favored (%)	96.07
Allowed (%)	3.93
Outlier (%)	0
